# Quantitative Three-Dimensional Imaging of Lipid, Protein, and Water Contents via X-Ray Phase-Contrast Tomography

**DOI:** 10.1371/journal.pone.0151889

**Published:** 2016-03-22

**Authors:** Marian Willner, Manuel Viermetz, Mathias Marschner, Kai Scherer, Christian Braun, Alexander Fingerle, Peter Noël, Ernst Rummeny, Franz Pfeiffer, Julia Herzen

**Affiliations:** 1 Department of Physics and Institute of Medical Engineering, Technische Universität München, Garching, Germany; 2 Institute of Forensic Medicine, Ludwig-Maximilians-Universität München, Munich, Germany; 3 Department of Diagnostic and Interventional Radiology, Technische Universität München, Munich, Germany; INIA, SPAIN

## Abstract

X-ray phase-contrast computed tomography is an emerging imaging technology with powerful capabilities for three-dimensional (3D) visualization of weakly absorbing objects such as biological soft tissues. This technique is an extension of existing X-ray applications because conventional attenuation-contrast images are simultaneously acquired. The complementary information provided by both the contrast modalities suggests that enhanced material characterization is possible when performing combined data analysis. In this study, we describe how protein, lipid, and water concentrations in each 3D voxel can be quantified by vector decomposition. Experimental results of dairy products, porcine fat and rind, and different human soft tissue types are presented. The results demonstrate the potential of phase-contrast imaging as a new analysis tool. The 3D representations of protein, lipid, and water contents open up new opportunities in the fields of biology, medicine, and food science.

## Introduction

Lipid, protein, and water are major components of biological tissues and other organic materials. Their concentrations play an important role in food science and medical research and diagnosis.

Accurate food compositional analysis has been a key factor in facilitating the food industry’s efforts to improve and standardize both its processes and products [1]. A strong relationship exists between the protein and fat percentages of meat and its final quality after processing [2–4]. In addition, fat and protein are also two main nutrients in dairy products, and variations in their contents influence various other properties, e.g., the sensations associated with yogurt or the firmness of cheese [5, 6].

In medicine, the concentrations of lipid, protein, and water are crucial parameters that reflect the progress of diseases. Their concentrations can provide insight regarding tissue functional changes associated with, e.g., the appearance, progression, and treatment of breast cancer. The water content of tumors is nearly two-fold greater than that of normal tissue, and the lipid content is reduced by about 45% [7]. The amount of collagen, a major stromal protein, increases in all abnormal breast tissues [8]. Hepatic steatosis is characterized by an abnormal accumulation of lipids within the liver. This is the earliest manifestation and hallmark of non-alcoholic liver disease that has a prevalence of approximately 20–30% in the adult population [9]. The disease may further progress into cirrhosis and liver failure. Several studies have shown that fibrosis, an excess deposition of extracellular matrix components including collagen, is a strong predictor of further progression to cirrhosis [10, 11].

Traditionally, laboratories have depended on a wide range of chemical analysis techniques to measure the levels of lipid, protein, and water. The Kjeldahl procedure for protein content and the Gerber or Rose-Gottlieb method for fat content are accurate and reliable, but are time-consuming and destructive methods for analysis [1]. The reference standard for tissue characterization in a clinical setting is histopathology. Biopsy specimens are taken from a patient, fixed in paraffin, and sectioned into thin slices. Various staining methods and subsequent visual assessments by light microscopy allow the pathologist to grade breast cancer or evaluate the degree of steatosis or stage fibrosis [12–14]. The disadvantages of histopathology are its invasiveness, observer-dependence, and the subjective estimation of the concentration of lipids or proteins, such as collagen, within the tissue.

Optical instruments are powerful tools for non-destructive and rapid compositional analysis. Spectroscopic techniques exploit the interaction of electromagnetic radiation with atoms and molecules to provide qualitative and quantitative chemical and physical information that is inherent within the frequency of the energy spectrum that is either absorbed or emitted [1]. Near-infrared reflectance spectroscopy enables the determination of the fat, protein, and water contents in meat under online industrial conditions to improve the efficiency of food manufacturing processes and automated quality control [15, 16]. Diffuse optical spectroscopy and Raman spectroscopy have generated considerable interest for non-invasive or minimally-invasive diagnostic methods and immediate diagnosis of a variety of breast pathologies as well as for monitoring the therapeutic response in tumor treatment [7, 8, 17].

The common factor among all the techniques mentioned thus far are that they are subject to sampling variability because only small fractions or portions of the sample under investigation can be analyzed at a given time. For this reason, quantitative three-dimensional (3D) imaging approaches, such as magnetic resonance imaging (MRI) or X-ray computed tomography (CT), are generally preferable for specific applications and examinations because they provide full spatial coverage.

Advanced MRI techniques allow the separate depiction of the water and fat components at any site of the human body [18]. These techniques exploit the difference in resonance frequencies between water and fat proton signals to quantitatively measure the proton density fat-fraction. This is a fundamental tissue property and a direct measure of fat content. MRI techniques currently under development have demonstrated high potential for the accurate detection and quantification of hepatic steatosis [19, 20]. When compared to histopathology, MRI is less time-consuming and allows the non-invasive evaluation of the entire liver parenchyma, resulting in a more complete assessment of liver-fat content.

CT is a widely used 3D imaging technology based on the differences in X-ray attenuation, and reflects the variations in density and compositional changes within the sample. The fat distribution in meat can be visualized and the degree of steatosis can be semi-quantitatively estimated by CT scans [19, 21]. In the absence of lipids, the contrast between various tissue types is very low, and no conclusions regarding water or protein concentration can be drawn. However, advances in X-ray detector technology offer new possibilities to utilize the full energy spectrum, and a recent study indicates that spectral CT measurements may be capable of characterizing the chemical composition of breast tissue in terms of water, lipid, and protein contents [22].

Another promising development for future X-ray applications is phase-contrast imaging. This approach relies on the refraction of X-rays rather than their attenuation. Several methods have been introduced to extract this additional contrast mechanism, which have demonstrated the high potential of this imaging approach for exploring low absorbing materials such as soft-tissue structures [23]. Among these methods is X-ray grating interferometry, a technique that can be successfully employed with laboratory X-ray sources, and that enables the quantitative imaging of electron densities within the sample [24–26]. The complementary information content opens new possibilities for enhanced tissue segmentation by combining conventional attenuation-contrast CT and phase-contrast CT [27–29]. This includes the assessment of lipid, protein, and water concentrations in each 3D tissue voxel, as we demonstrate in this paper.

## Results

**Fig 1:**
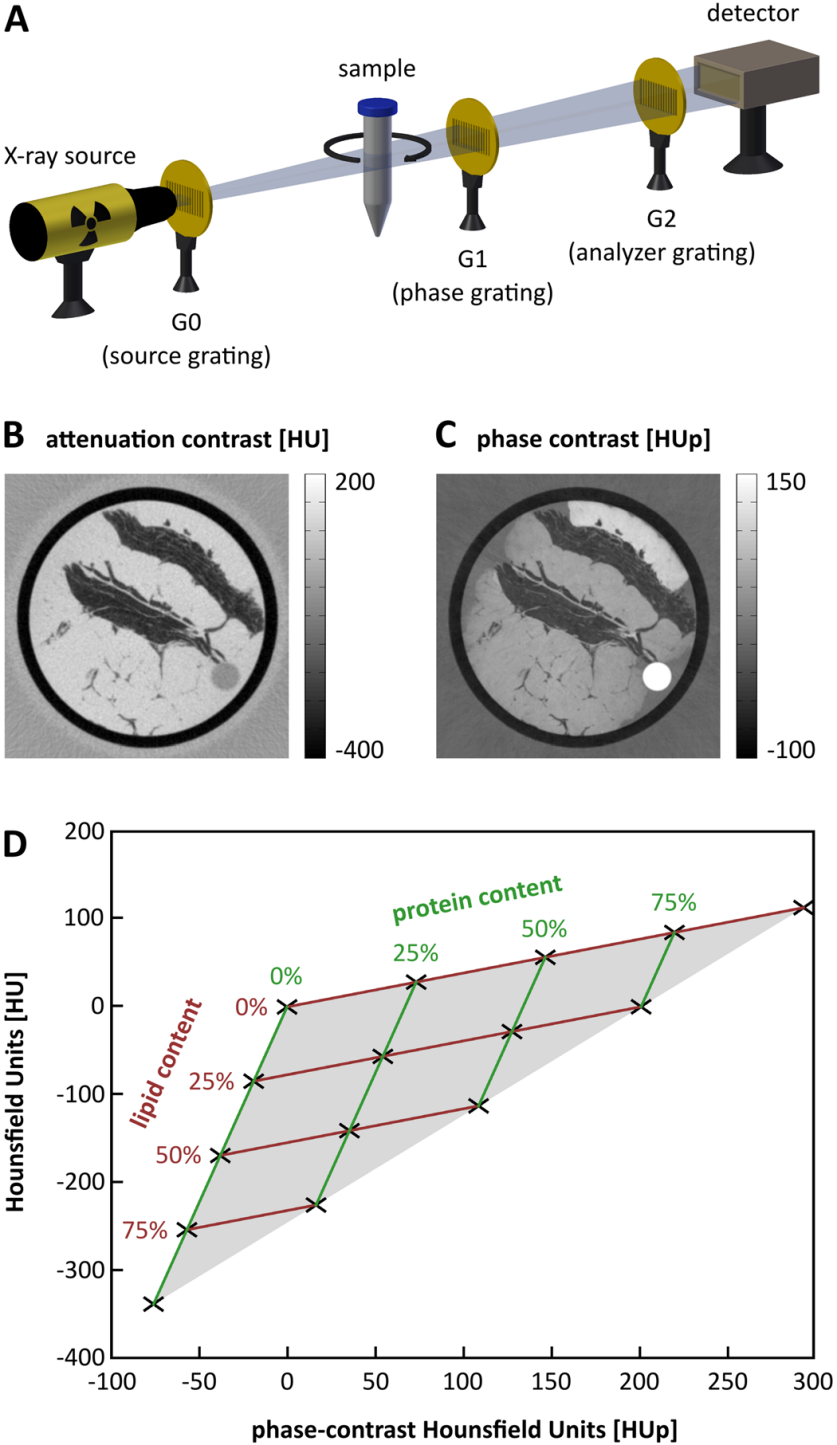
Concept illustration for three-dimensional (3D) characterization of lipid, protein, and water concentrations. (A) Schematic drawing of the three-grating Talbot-Lau interferometer used for the measurements in this study. (B and C) Exemplary tomographic imaging results of a porcine fat and rind sample in attenuation and phase contrast, respectively. The obtained 3D datasets represent the distribution of the linear attenuation coefficient *μ* and the refractive index decrement *δ* within the object and can be converted to quantitative Hounsfield units (HU and HUp). (D) Theoretically calculated HU and HUp values for fictive mixtures of varying protein and lipid concentrations. The results span a triangle, and every (experimentally evaluated) HU-HUp-pair can be assigned to exactly one specific protein and lipid content.

A typical grating-based phase-contrast imaging system operated in a laboratory environment combines a standard X-ray tube, a Talbot-Lau interferometer consisting of three gratings, and an X-ray detector, as schematically illustrated in Fig 1A [25]. The first grating comprises attenuating gold structures, and is placed behind the source to create an array of individually coherent but mutually incoherent sources. This allows for interference effects induced by the second grating. The resulting interference pattern can then be analyzed by another gold grating mounted directly in front of the detector to draw conclusions based on beam attenuation and refraction caused by the examined samples.

When recording an object from different angular directions in a tomographic measurement, the conventional attenuation-based 3D volume and a spatially fully co-registered phase-contrast dataset can be reconstructed. The technique is quantitative and enables the assignment of Hounsfield units (HU)—based on linear attenuation coefficients *μ*—to the 3D voxels as routinely used in clinical diagnostics. In phase contrast, the electron density distribution within the object can be retrieved and similar phase-contrast Hounsfield units (HUp) can be defined [30]. Exemplary images in both contrast modalities showing an axial slice through a porcine fat and rind sample are displayed in Fig 1B and 1C. The complementarity of the imaging signals becomes clearly apparent in the collagen-rich rind (uppermost feature), which provides high values in phase contrast but is rather unremarkable in attenuation contrast. The latter, on the other hand, achieves good contrast for fatty tissue (dark regions), as is commonly known from medical CT imaging. This implies that an advanced tissue characterization with respect to protein or lipid content might be possible when combining both the contrast modes.

For a more systematic investigation of the issue, theoretical (conventional) HU and (phase-contrast) HUp were calculated for fictive tissues with varying protein and lipid volume fractions. The remaining unaccounted for volume fraction of up to 100% was assumed to be water. Tabulated elemental compositions and protein and lipid densities required for the calculation were taken from the literature [31]. The results are presented in the HU-HUp-scatter plot in Fig 1D. In the figure, the HUp values associated with phase contrast are plotted on the x-axis and the corresponding attenuation-based HU values are plotted on the y-axis. Obviously, increasing lipid content has a comparably larger effect on the attenuation signal, whereas the phase-contrast signal is more affected by the protein concentration. Therefore, the values of all considered fictive tissue compositions form a triangle in which every HU-HUp-pair can be associated with exactly one distinct triplet of protein, lipid, and water content (*p*/*l*/*w*). Because of the linear relationship of HU and HUp values with protein and lipid, a vector decomposition can be applied to determine their respective concentrations. The water content can then be obtained according to *w* = 1 − *p* − *l*. The formulas used in the calculation of HU and the vector decomposition are given in the Materials and Methods section.

**Fig 2:**
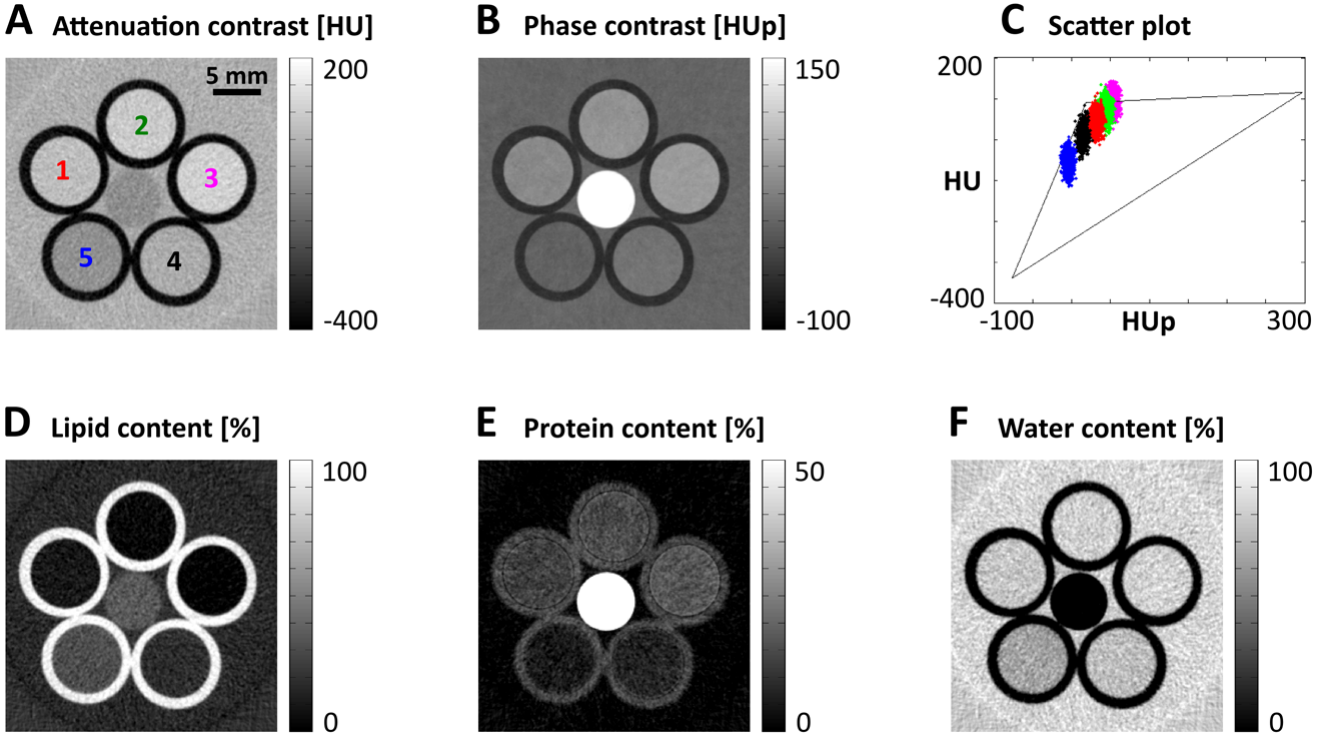
Results from a test phantom consisting of the various dairy products listed in Table 1. (A and B) Exemplary tomographic attenuation-contrast and phase-contrast images, respectively, displaying the five vials filled with dairy products (1–3: cream cheese, 4 and 5: sour cream) circularly arranged around a polymethylmethacrylate rod. (C) The quantitative HU and HUp values of each dairy product arranged around a certain point in the corresponding HU-HUp-scatterplot according to its protein and lipid content. (D–F) Images reflecting the lipid, protein, and water contents of the dairy products generated by the vector decomposition of the attenuation-contrast and phase-contrast data.

A first evaluation of the approach was performed using a custom-built phantom consisting of five vials filled with various dairy products (3 types of cream cheese and 2 types of sour cream). The contents of lipid (ranging from 0.3% to 33.8%), protein (ranging from 2% to 9.3%), carbohydrates, and water, as mentioned on the packages, were converted into volume fractions. The resulting values are listed in Table 1. Information regarding the imaging setup and scan parameters can be found in the Material and Methods section. Exemplary images in attenuation and phase contrast recorded at a tube voltage of 40 kV are shown in Fig 2A and 2B, respectively. HU and HUp values for a region-of-interest (ROI) containing 20×20×4 voxels from each product are plotted in Fig 2C. Color-coding reveals that all points corresponding to a given product are grouped around a specific point in the HU-HUp-scatter plot. The dimensions of the point clouds are caused by image noise, which was around 18 HU and 3.5 HUp in the measurement.

**Table 1:** Experimental lipid, protein and water concentrations (exp.) of the examined dairy products (dp) in comparison to the contents stated by the manufacturers (pack.).

	dp 1	dp 2	dp 3^a^	dp 4	dp 5
lipid content [%]					
pack.	0.3	4.7	11.0	16.8	33.8
exp.	0.6	5.0	11.0	19.9	34.5
protein^b^ content [%]					
pack. (prot.)	9.3	8.1	7.1	2.1	2.0
pack. (carb.)	3.0	2.7	2.7	3.1	2.1
pack. (prot.+carb.)	12.3	10.8	9.8	5.2	4.1
exp.	14.0	12.1	9.8	6.3	4.5
water content [%]					
pack.	87.4	84.5	79.2	78.0	62.1
exp-	85.4	83.0	79.2	73.8	61.0

^a^ The dairy product with mid-lipid and mid-protein content was taken for calibration of the vector decomposition.

^b^ Carbohydrates (carb.) stated in the nutrition tables of the packages were counted to the protein content (prot.) for the analysis.

The end points of the two vectors required for the decomposition were chosen to be the calculated HU-HUp-values of 100% protein and 100% lipid. However, their joint starting point was not fixed at 0 HU and 0 HUp (100% water) to allow for a more flexible data analysis, which might be necessary to account for influences such as density changes due to organic binders or high absorbing trace elements within the products. Instead, a dairy product with mid-range lipid and protein contents was used for the calibration, and the vector starting points were adjusted to achieve accordance between the analysis results and the product’s nutrition table. Carbohydrates (between 2.1% and 3.1%) were added to the protein contents in this context and for the validation of the analysis results of the other four products. Decomposed lipid, protein, and water images of the phantom are displayed in Fig 2C–2E. The lipid image appears generally noisier than the protein image (about 4.3% versus 1.9%) because of the major contribution of the attenuation data to image formation. Mean protein, lipid, and water contents (from the same ROIs as before) are summarized in Table 1. The experimental results show good agreement with the contents stated by the manufacturers. The deviations for the lipid content are only between 0.3% and 0.7% in volume fraction for all dairy products but one, which gave a content of 19.9% compared to 16.8%. The protein concentrations differ in the range of 0.4–1.7% from the actual values. The largest discrepancies (up to 4.2%) can be observed for the water content.

**Fig 3:**
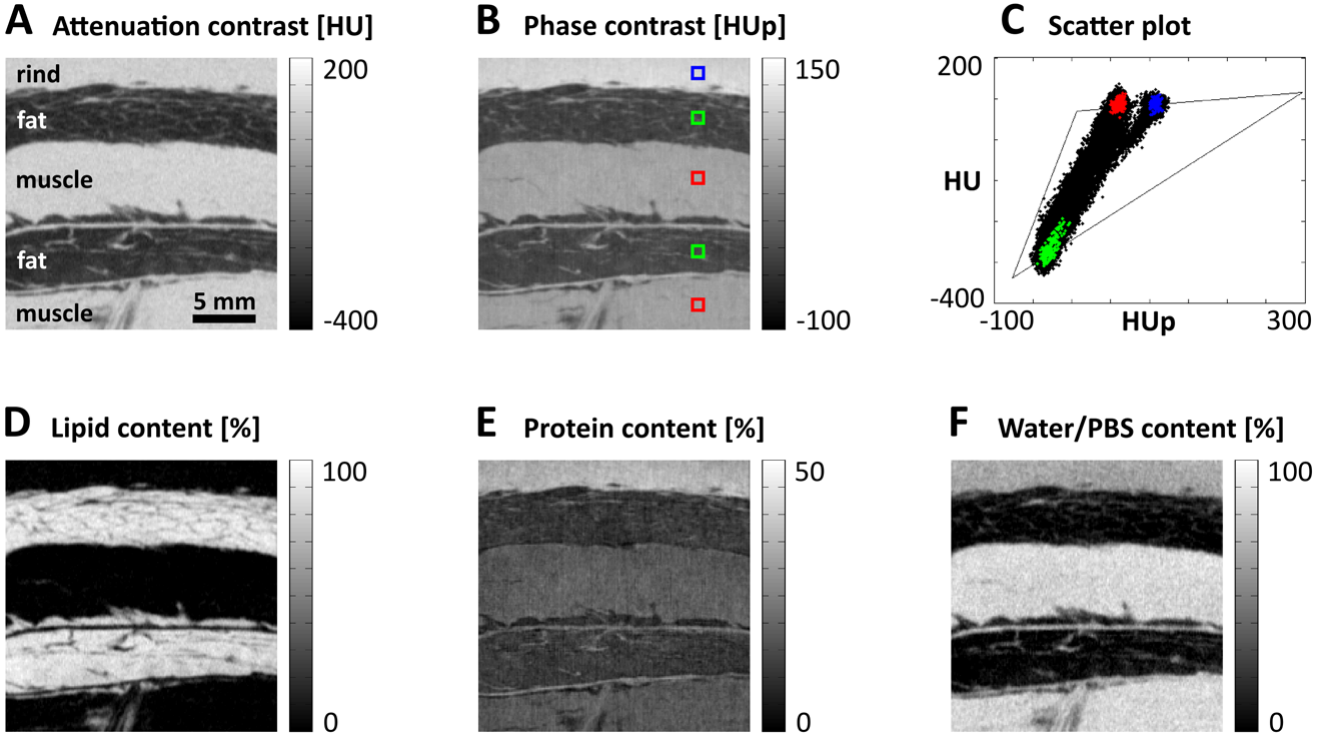
Vector decomposition of biological soft tissues using the example of porcine fat and rind. (A and B) Tomographic attenuation-contrast and phase-contrast imaging results, respectively, of a section covering rind, fat, and muscle. (C) Values of the regions-of-interest marked in the different tissue types are clearly separated in the HU-HUp-scatterplot. (D–F) Decomposed lipid, protein, and water images reveal the high lipid content of the fat, the increased protein (collagen) content in the rind, and the high water concentration of the muscle.

As a first biological example, the sample of porcine fat and rind mentioned earlier was examined more closely. The sample was placed in a cylindrical falcon tube (3 cm diameter) filled with phosphate buffered saline (PBS) and cooled to 5°C during the measurement. Attenuation-contrast and phase-contrast images of a section containing the three main tissue types, rind, muscle and fat, are presented in Fig 3A and 3B, respectively. Small ROIs marked in the rind (blue), muscle (red), and fat (green) can be allocated to distinct points in the HU-HUp-scatter plot (Fig 3C) reflecting the different protein and lipid contents of the tissues. To conduct the decomposition, the measured HU and HUp values of the surrounding physiological PBS solution were taken as the starting point of the vectors. The end points were equivalent to those used in the phantom study. The resulting lipid, protein, and water images are displayed in Fig 3D–3F. The fatty tissue stands out in the lipid image, whereas rind and muscle scarcely contain any lipids. The higher amount of collagen in the rind is evident in the protein image. Some filaments at the interfaces between fat and muscle are also visible as brighter protein signals. The highest water content is found in the muscle.

**Fig 4:**
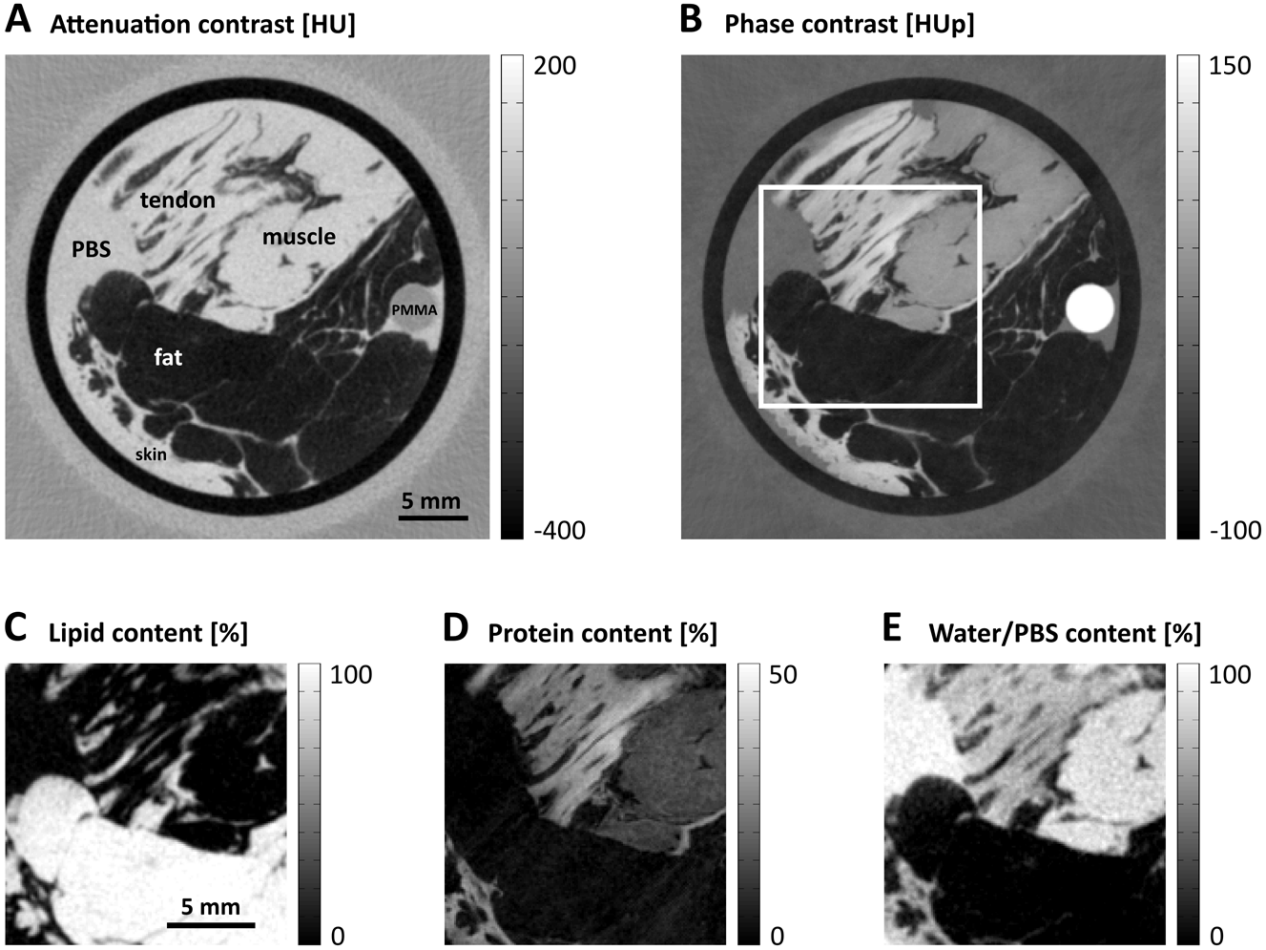
Lipid, protein, and water analysis of different human soft tissue types. (A and B) Axial tomographic slices in attenuation and phase contrast, respectively, through the measured plastic cylinder containing tendon, muscle, fat, and skin tissues. (C–E) Decomposed images of the region marked by the white box in (B).

The second biological sample comprised four human soft tissue types, i.e., adipose/fatty tissue, muscle, skin, and tendon. The tissues were placed in a falcon tube with PBS and cooled during the scan. Fig 4A and 4B exhibit the obtained attenuation-contrast and phase-contrast imaging results, respectively, for the four tissue types within the plastic cylinder. Panels C–E show the area marked by the white box in Fig 4B decomposed into its lipid, protein, and water contents. The vectors for the decomposition were again defined by the measured HU and HUp values of the PBS and the calculated values for 100% protein and 100% lipid. Small ROIs of fat, muscle, and tendon were selected to quantify their respective lipid, protein, and water concentrations. The experimental results are listed in Table 2 together with the values reported in the literature for comparison [31]. Despite the great variety of a tissue’s composition in general, the experimental and quoted protein contents are very similar in all the three cases with differences of only 0.3% to 1.1% in volume fraction. The lipid content of adipose is increased compared to the tabulated content. As a consequence, the water content was determined to be −2.6%. The negative value indicates that a slight modification of the vectors chosen for the decomposition is necessary to cover all the data points.

**Table 2:** Quantitative analysis of the lipid, protein, and water contents (exp.) of human tissue types in comparison to values tabulated in the literature (tab.) [31].

	fat	muscle	tendon
lipid content [%]			
exp.	99.2	-0.8	0.1
tab.	76.7	4.9	1.2
protein content [%]			
exp.	3.4	15.3	29.3
tab.	3.1	15.7	30.4
water content [%]			
exp.	-2.6	85.5	70.6
tab.	20.2	79.4	68.2

## Discussion

The high sensitivity of phase-contrast CT to detect small differences in electron density offers significant potential for application in biomedical research. The complementary information provided in addition to conventional attenuation-based imaging allows for enhanced tissue characterization. As demonstrated in this proof-of-concept study, protein, lipid, and water contents within each 3D voxel can be quantified by applying a vector decomposition for the data obtained with both contrast modalities.

Further investigations should address the optimum choice of the utilized vectors and the effect of trace elements on the analysis results. Trace amounts of heavier elements can presently increase the attenuation signal and affect the extracted lipid content. In the presented phantom study, this issue was mostly addressed using a single dairy product as a calibration substance and accordingly adjusting the vector starting points. For the biological examples, the consideration of the physiological PBS solution in the definition of the vector starting points accounted for heavier elements. The presence and differences of the manifold types of proteins and lipids have not been examined more closely yet and might have an impact on the correct choice of the vectors. Based on our promising but preliminary results, we therefore envision a comprehensive study alongside with a statistically meaningful analysis in near future, to exactly validate the diagnostic specificity of this lipid, protein, and water decomposition technique on a more quantitative basis.

One limitation of the proposed method is the loss of complementarity given by attenuation and phase contrast imaging with increasing X-ray energies, which to its end might decrease the accuracy when determining protein and lipid contents. At energies above 50 keV, Compton scattering is the dominating attenuation process for materials with effective atomic numbers below 8 (including most biological soft tissues). Under these conditions, both the attenuation and the phase signal are proportional to the electron density [32]. In this case, vector decomposition might prove useful for other applications, e.g., the specification of contrast agents in blood or calcium content in bones or teeth.

For the energy range employed in this study, the decomposition of protein, lipid, and water contents is especially promising for preclinical imaging. A first small-animal phase-contrast CT scanner with rotating gantry is currently under construction [33]. There are as yet various challenges to overcome for performing dose-compatible tomographic scans of mice, but in-vivo radiographic imaging is already feasible [34]. Another interesting direction to pursue with phase-contrast imaging in the lower energy regime is virtual 3D histology [35, 36]. The spatial distribution of proteins and lipids may enable enhanced identification of abnormalities such as early-stage tumors or fibrosis within biopsy samples. A further development that could benefit from advanced tissue characterization in terms of protein and lipid concentrations is breast CT. The feasibility and benefit of such a device (attenuation-based) is presently under discussion, and several preliminary examinations indicate a potential for added value by including phase contrast [37–39]. In addition, the approach may open up new perspectives in the field of food science.

Realization of phase-contrast imaging for any one of these applications is expected to clearly outperform corresponding dual or spectral energy solutions with respect to protein and lipid analysis. Attenuation-based alternatives require Compton scattering dominated high energies to obtain electron density information equivalent to that provided by phase contrast. This inevitably involves low image quality of low absorbing objects, such as small tissue samples, and very high noise levels in the decomposed protein images.

## Materials and Methods

### Grating-based phase-contrast imaging setup

The phase-contrast imaging system used for the experiments is located at the Technische Universität München, Germany. It consists of a rotating molybdenum anode X-ray tube, a Talbot-Lau grating interferometer, and a photon-counting detector (Pilatus II, Dectris, Switzerland). The three gratings employed in the interferometer were fabricated at the Karlsruhe Institute of Technology (KIT), Germany. All the gratings were made of gold with periods of 5.4 *μ*m. Two of the gratings act as transmission gratings with structure heights of about 70 *μ*m. They are installed behind the source and in front of the detector, respectively, with a distance of 170 cm between the two. A phase grating of 5.2 *μ*m height is directly placed in the middle and introduces a phase shift of *π* to X-rays of 27 keV. The rotation stage is mounted close to the phase grating providing a sample magnification of 1.7. This results in a field of view of 4×2 cm^2^ and an effective pixel size of 100×100 *μ*m^2^. The phase stepping procedure required to extract the phase information is performed by a mechanical nano-converter driven by a precision motorized actuator. The visibility of the interferometer is about 0.25.

### Sample preparation and measurements

For the phantom study, five dairy products—different types of cream cheese and sour cream—were purchased from random manufacturers and placed in small cylindric vials of 5 mm diameter, which were arranged circularly around a polymethylmethacrylate (PMMA) rod. The porcine fat and rind was obtained from a local butcher, and the human tissue samples were excised at the Institute of Forensic Medicine (Ludwig-Maximilians-Universität München, Germany) and was approved by the local Ethics Committee of the Faculty of Medicine of the Technische Universität München. The review board waived the need for consent. All the tissue samples were placed in *ϕ*3 cm plastic cylinders filled with a physiological PBS. The samples were submerged in a water bath during the measurements to reduce beam hardening and avoid phase wrapping artifacts [40]. In the case of the soft tissue samples, the water was cooled to a temperature of 5°C to slow down the decay process. 800 projections over 360° were acquired at a tube voltage of 40 kV and a current of 70 mA for each tomographic scan. A projection comprising 11 phase step images recorded with exposure times of 3 s was processed by a (weighted) least-squares fit to obtain phase and attenuation information. A standard filtered backprojection algorithm was applied to reconstruct the 3D volumes. For the phase-contrast images, the filter function was replaced by an imaginary Hilbert filter [27]. Taking setup-dependent factors into consideration, the distribution of the linear attenuation coefficient *μ* and the refractive index decrement *δ* within the samples (relative to the surrounding water) were determined from the attenuation-contrast and phase-contrast datasets, respectively [26].

### Quantitative attenuation-contrast and phase-contrast HU

In clinical routines, quantitative HU simplify diagnostics using X-ray CT imaging. These units are defined by

$$\text{HU}=\frac{\mu_{\text{tissue}}-\mu_{\text{water}}}{\mu_{\text{water}}-\mu_{\text{air}}}\cdot 1000,$$ (1)

where *μ*_tissue_, *μ*_water_ and *μ*_air_ are the linear attenuation coefficients of the associated tissue, water, and air, respectively. Similar HUp can be assigned in phase-contrast imaging by replacing the respective linear attenuation coefficients *μ* by the corresponding refractive index decrements *δ* [30]:

$$\text{HUp}=\frac{\delta_{\text{tissue}}-\delta_{\text{water}}}{\delta_{\text{water}}-\delta_{\text{air}}}\cdot 1000.$$ (2)

To derive HU and HUp values from the experimental data, the contribution of air was neglected and the theoretical values of *μ*_water_ and *δ*_water_ were calculated. A PMMA rod was used as the calibration material in every measurement to identify the respective effective energies of the polychromatic spectrum [39]. For a mixture of density *ρ*_mix_ consisting of several chemical elements *i*, the linear attenuation coefficient *μ*_mix_ is given by

$$\mu_{\text{mix}}=\rho_{\text{mix}}\cdot \sum w_{i}\cdot \frac{\mu_{i}}{\rho_{i}},$$ (3)

where *w*_*i*_, *μ*_*i*_ and *ρ*_*i*_ are the weight fraction, linear attenuation coefficient and density of the *i*-th element, respectively. The corresponding refractive index decrement *δ*_mix_ can be determined according to

$$\delta_{\text{mix}}=\frac{r_{0}h^{2}c^{2}}{2\pi E^{2}}\cdot \rho_{mix}\cdot \sum \left(w_{i}\cdot N_{A}/A_{i}\right)\cdot Z_{i}$$ (4)

using the classical electron radius *r*_0_, the speed of light *c*, (effective) energy *E* of the X-rays, Avogadro’s number *N*_*A*_, and the elements’ atomic masses *A*_*i*_ and atomic numbers *Z*_*i*_[26]. The formula is valid for energies far above any absorption edges as is the case in the present study. For the calculations of HU and HUp values of mixtures with varying protein (p), lipid (l), and water (w) concentrations, the densities *ρ*_mix_ and weight fractions *w*_*i*,mix_ were obtained by

$$\rho_{\text{mix}}=p\cdot \rho_{p}+l\cdot \rho_{l}+w\cdot \rho_{w}$$ (5)

and

$$w_{i,\text{mix}}=\frac{\rho_{p}}{\rho_{\text{mix}}}\cdot w_{i,p}+\frac{\rho_{l}}{\rho_{\text{mix}}}\cdot w_{i,l}+\frac{\rho_{w}}{\rho_{\text{mix}}}\cdot w_{i,w}.$$ (6)

The densities *ρ*_p_, *ρ*_l_, *ρ*_w_ and elemental compositions in weight fractions *w*_*i*,p_, *w*_*i*,l_, *w*_*i*,w_ of protein, lipid and water were taken from tabulated data [31]. The volume fractions *p*, *l*, *w* of protein, lipid and water were always chosen to represent the entire mixture (*p* + *l* + *w* = 1).

### Vector decomposition in lipid, protein, and water contents

Because of the linear increase and decrease in the HU and HUp values with increasing protein and lipid concentrations, every possible mixture can be associated to exactly one HU-HUp-pair described by

HUpHU=w→+p·p→-w→+l·l→-w→=w→+p·a→+l·b→, (7)

where $\vec{w}$, $\vec{p}$, and $\vec{l}$ represent the HU-HUp-pairs of 100% water, 100% protein, and 100% lipid, respectively. Conversely, the contents of protein *p* and lipid *l* in a mixture/tissue can be evaluated by the vector decomposition of the experimentally determined HU and HUp:

$$p=\frac{\vec{b}\left(1\right)\cdot \left[\text{HU}-\vec{w}\left(2\right)\right]-\vec{b}\left(2\right)\cdot \left[\text{HUp}-\vec{w}\left(1\right)\right]}{\vec{a}\left(2\right)\cdot \vec{b}\left(1\right)-\vec{a}\left(1\right)\cdot \vec{b}\left(2\right)}$$ (8)

and

$$l=\frac{\vec{a}\left(1\right)\cdot \left[\text{HU}-\vec{w}\left(2\right)\right]-\vec{a}\left(2\right)\cdot \left[\text{HUp}-\vec{w}\left(1\right)\right]}{\vec{a}\left(1\right)\cdot \vec{b}\left(2\right)-\vec{a}\left(2\right)\cdot \vec{b}\left(1\right)}.$$ (9)

The missing water fraction is then obtained by

w=1-p-l. (10)

Pure water (*w* = 1) has, by definition, 0 HU and 0 HUp ($\vec{w}=0$). To allow the compensation of distorting effects on the analysis results (e.g., caused by the presence of heavier elements), the vector $\vec{w}$, and, thus, the shared starting point of the two vectors $\vec{a}$ and $\vec{b}$ were individually adjusted for the performed vector decompositions. In the phantom analysis, the dairy product with mid-range protein and lipid contents was used for calibration resulting in w→=(14.5HUp90.5HU). For the soft-tissue samples, $\vec{w}$ was adapted to the experimental HU and HUp values of the physiological PBS (6.5 HUp and 70 HU). The end points $\vec{p}$ and $\vec{l}$ of the vectors $\vec{a}$ and $\vec{b}$ were held fixed in all the three examples and chosen to match the calculated values for 100% protein (p→=(296.8HUp115.1HU)) and 100% lipid (l→=(-76.1HUp-338.6HU)).
